# Which is your choice for prolonging the analgesic duration of single-shot interscalene brachial blocks for arthroscopic shoulder surgery? intravenous dexamethasone 5 mg vs. perineural dexamethasone 5 mg randomized, controlled, clinical trial

**DOI:** 10.1097/MD.0000000000003828

**Published:** 2016-06-10

**Authors:** Eun Hee Chun, Youn Jin Kim, Jae Hee Woo

**Affiliations:** Department of Anesthesiology and Pain Medicine, School of Medicine, Ewha Womans University, Seoul, Korea.

**Keywords:** analgesia, analgesics anti-inflammatory, anesthetic techniques, anesthetics local, brachial plexus, postoperative, regional, ropivacaine, steroid

## Abstract

The aim of this study was to compare the effect of intravenous (I.V.) dexamethasone with that of perineural dexamethasone on the prolongation of analgesic duration of single-shot interscalene brachial plexus blocks (SISB) in patients undergoing arthroscopic shoulder surgery. We performed a prospective, randomized, double-blind, placebo-controlled study. Patients undergoing elective arthroscopic shoulder surgery with ultrasound-guided SISB were enrolled and randomized into 2 groups. A total volume of 12 mL of the study drug was prepared with a final concentration of 0.5% ropivacaine. In the I.V. group, patients received SISB using ropivacaine 5 mg mL^−1^ with normal saline (control) with dexamethasone 5 mg I.V. injection. In the perineural group, patients received SISB using ropivacaine 5 mg mL^−1^ with dexamethasone 5 mg, with normal saline 1 mL I.V. injection. The primary outcome was the time to the first analgesic request, defined as the time between the end of the operation and the first request of analgesics by the patient. The secondary outcomes included patient satisfaction scores, side effects, and neurological symptoms. Patients were randomly assigned to 1 of the 2 groups using a computer-generated randomization table. An anesthesiologist blinded to the group assignments prepared the solutions for injection. The patients and the investigator participating in the study were also blinded to the group assignments. One hundred patients were randomized. Data were analyzed for 99 patients. One case in the I.V. group was converted to open surgery and was therefore not included in the study. Perineural dexamethasone significantly prolonged analgesic duration (median, standard error: 1080 minutes, 117.5 minutes) compared with I.V. dexamethasone (810 minutes, 48.1 minutes) (*P* = 0.02). There were no significant differences in side effects, neurological symptoms, or changes in blood glucose values between the 2 groups. Our results show that perineural dexamethasone 5 mg is more effective than I.V. dexamethasone 5 mg with regard to analgesic duration of SISB for arthroscopic shoulder surgery.

## 1 Introduction

Single-shot interscalene brachial plexus blocks (SISB) are widely used for postoperative pain control, but the analgesic duration of the procedure is time-limited compared with continuous interscalene brachial plexus blocks. The development of multimodal perineural analgesia is expected to extend the duration of motor and sensory blocks, and to reduce both side effects and the cost of continuous blocks. A lower incidence of adverse effects and improved analgesia with reduced opioid consumption have been observed with multimodal analgesia techniques in arthroscopic shoulder surgery.^[1]^ Furthermore, it is possible to meet the patient's needs without the insertion of a perineural catheter.

Dexamethasone is a representative adjunct for antiemesis as well as postoperative pain control in multimodal strategies.^[2,3]^ A meta-analysis has confirmed that perineural dexamethasone with local anesthetics prolongs the effects of brachial plexus block (BPB).^[4]^ However, the perineural administration of dexamethasone is an “off-label” use, and there are no pharmacokinetic data on dexamethasone using the perineural route. In addition, no guidelines exist regarding the optimal dose of perineural dexamethasone with local anesthetics in BPB.

Interestingly, several studies have reported that dexamethasone prolongs the analgesic duration of SISB regardless of the route of administration.^[5,6]^ However, the equivalence of intravenous (I.V.) dexamethasone to perineural dexamethasone has not yet been demonstrated. The determination of the route and dose of dexamethasone depend on the anesthesiologist's experience and the available underpowered data regarding effective multimodal analgesia following arthroscopic shoulder surgery. The aim of the present study was to compare the effect of I.V. dexamethasone with that of perineural dexamethasone on the prolongation of analgesic duration in patients receiving SISB.

## 2 Methods

This study was approved by the Ewha Womans University Mokdong Hospital Institutional Review Board (2014-12-026-001), and written informed consent was obtained from all patients. The trial was registered at the Clinical Trial Registry of Korea (KCT0001473). Patients (aged 20–80 years, American Society of Anesthesiologists [ASA] physical status I–II) undergoing elective arthroscopic shoulder surgery were included. Exclusion criteria included any neuropathy, coagulopathy, respiratory diseases, systemic steroid use or chronic opioid use, and uncontrolled diabetes mellitus (DM).

The primary outcome was the time to the first analgesic request, defined as the time between the end of the operation and the first request of analgesics by the patient. Secondary outcomes included analgesic administration, pain scores, and incidences of motor block, numbness, and side effects for 2 days postoperative.

From our prior experience and evidence from previous reports,^[7,8]^ we anticipated an analgesic duration of 24 hours with ropivacaine and dexamethasone 5 mg, with a 6-h difference between the groups representing a clinically significant difference. Thus, a sample size of 47 patients (power = 80%, α = 0.05) per group was determined to be appropriate. Taking potential dropout into account, we planned to recruit 50 patients per group.

Patients were randomly assigned to 1 of the 2 groups using a computer-generated randomization table. In the I.V. group, patients received an injection of 12 mL of a solution containing of ropivacaine 0.75% (60 mg) and 4 mL of 0.9% saline. In the perineural group, patients received an injection of 12 mL of a solution containing 8 mL of ropivacaine 0.75% (60 mg), 3 mL of 0.9% saline, and dexamethasone 5 mg for SISB. An anesthesiologist blinded to the group assignments prepared the solutions for injection. The patients and the investigator participating in the study were also blinded to the group assignments.

After measurement of blood pressure (BP), heart rate (HR), and oxygen saturation by pulse oximetry (SpO_2_), fentanyl (25–50 μg) and midazolam (1–3 mg) were administered intravenously. SISB was performed by an experienced anesthesiologist under ultrasound guidance (M-Turbo; SonoSite, Inc., Bothell, WA). After sterile skin preparation, the superior trunk or nerve roots located between the anterior and middle scalene muscles were identified using a linear probe (5–12 MHz). We used the in-plane method, and a 22-gauge, 50-mm, insulated needle (UniPlex NanoLine, PAJUNKGmbH Medizintechnologie, Geisingen, Germany) was advanced toward the roots or superior trunk within the sheath. At the time of the SISB, 1 mL of a solution containing normal saline for the perineural group or dexamethasone 5 mg for the I.V. group was injected intravenously.

General anesthesia was induced with thiopental sodium 4 mg kg^−1^, fentanyl 1–2 μg kg^−1^, and rocuronium 0.6 mg kg^−1^. Anesthesia was maintained using an oxygen/air mixture with sevoflurane [1.0–1.5 minimum alveolar concentration (MAC) levels] with bispectral index (BIS) values of 40 to 60. All operations were performed by a single surgeon. Finger stick blood glucose levels were checked before the beginning of the block procedure (baseline) and before discharge from the recovery room. In the recovery room, patients were assessed by an anesthesiologist for pain score (using the numerical rating scale [NRS]; 0, no pain; 10, most severe pain imaginable), motor block, numbness, and complications or side effects. The existence of phrenic nerve palsy was confirmed by a chest x-ray of postoperative evaluations by an orthopedic surgeon. Patients were administered I.V. tramadol 50 mg, up to 150 mg a day, when NRS scores were 3 or higher. If this analgesia was insufficient, I.V. ketorolac 30 mg was administered to patients up to 3 times a day.

A doctor blinded to the group assignments assessed the patients at 6, 12,18, 24, and 48 hours from the time of arrival in the recovery room, and recorded the time to the first analgesic request as well as pain using the NRS. The pain relief satisfaction score of patients was evaluated using a 3-point scale (0 = unsatisfied, 1 = uncertain, 2 = satisfied) at 48 hours.

Data were analyzed using SPSS (ver.18.0, Chicago, IL). The Mann–Whitney *U* test was performed to compare continuous variables between groups after assessment for normality. Data are presented as mean ± standard deviation (SD) or as median and standard error, as appropriate. Categorical variables were analyzed using the χ^2^ or Fisher's exact tests. Blood glucose values were analyzed using the Mann–Whitney *U* test and Wilcoxon signed-rank test. The time to the first analgesic request was analyzed using Kaplan–Meier survival analysis with the log-rank test. A *P* value of <0.05 was considered statistically significant.

## 3 Results

**Figure 1:**
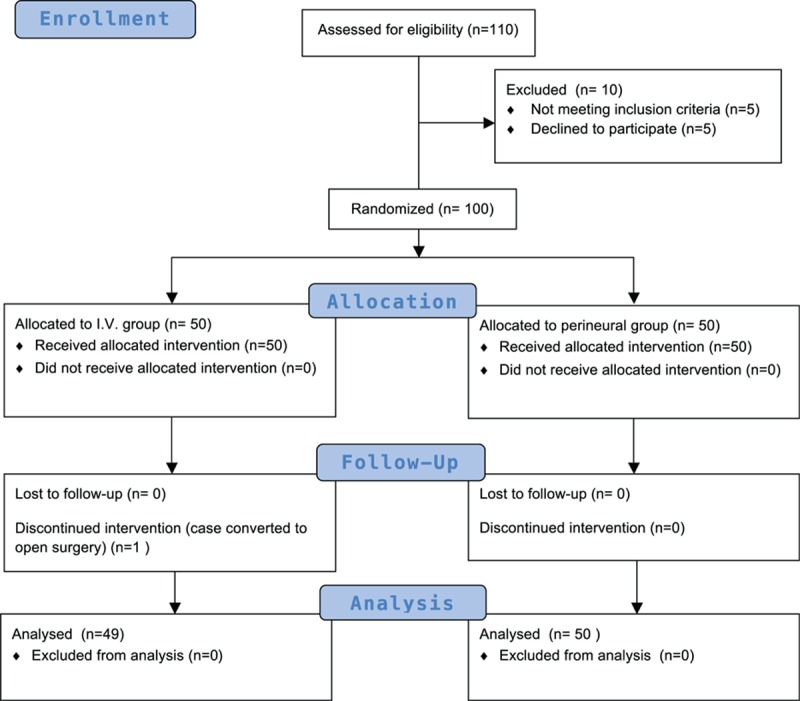
Flowchart of patient enrollment.

The flowchart of patient enrollment in the present study is shown in Fig. 1. A total of 110 patients were screened for participation in this study; 100 patients were randomized, and final data were analyzed for 99 patients. One case in the I.V. group was converted to open surgery and was therefore not included in the study. Patient demographic and clinical features did not differ between the 2 groups (Table 1). Eleven patients in the I.V. group and 6 in the perineural group had DM. All were well-controlled DM patients whose glycated hemoglobin (HbA1c) values were reported to be within normal limits, and no patients showed evidence of DM neuropathy.

**Table 1:**
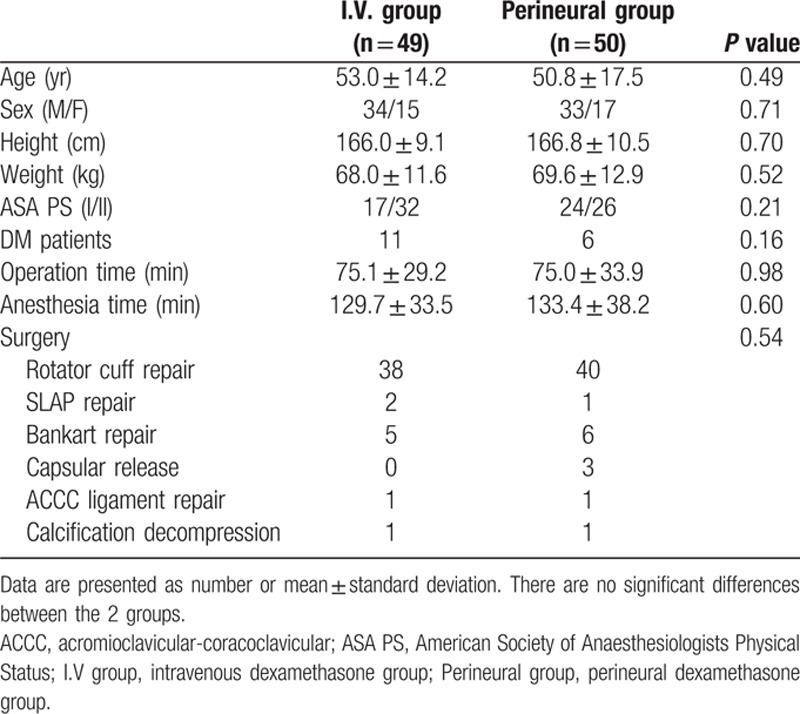
Demographic and clinical features of patients.

**Figure 2:**
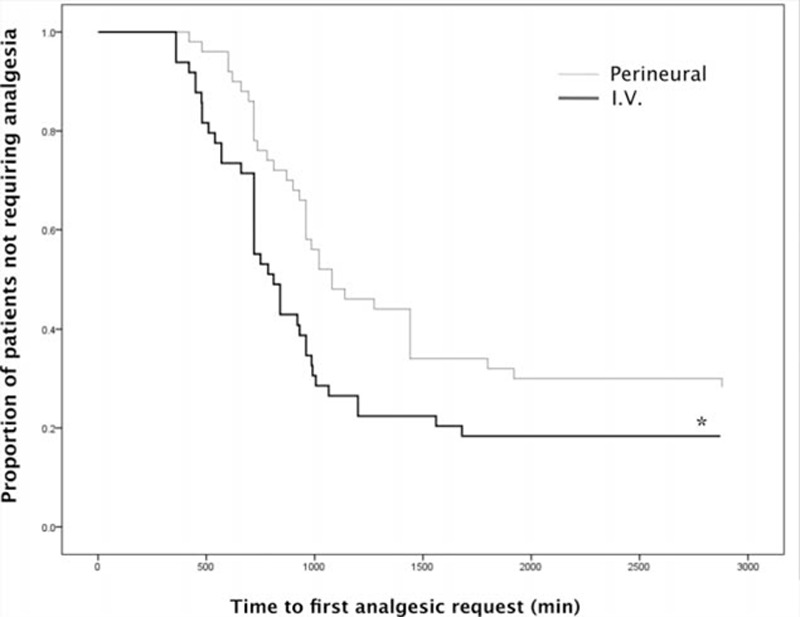
Kaplan–Meier plot describing analgesic duration in the study groups. ^∗^: Perineural dexamethasone significantly prolonged analgesic duration compared with I.V. dexamethasone (*P* = 0.02).

The definition of “median analgesia time” was the time to first analgesic request in >50% of patients. The median analgesia time (median, standard error) was 810 minutes, 48.1 minutes in the I.V. group, and 1080 minutes, 117.5 minutes in the perineural group. Perineural dexamethasone significantly prolonged analgesic duration compared with I.V. dexamethasone (*P* = 0.02) (Fig. 2).

Table 2 shows postoperative pain-related outcomes. There were no significant differences in baseline and postoperative NRS between the 2 groups. Within the first 24 hours, which is thought to be the most painful period, the number of patients not requiring analgesics was 22 in the perineural group and 11 in the I.V. group (*P* = 0.03). There were no significant differences in the use of tramadol or ketorolac. The patients’ pain relief satisfaction scores were significantly better in the perineural group compared to the I.V. group (*P* = 0.02). Block characteristics and adverse effects are shown in Table 3. There were no significant differences in the incidence of motor block, numbness, or other events including diaphragm elevation, hoarseness, Horner syndrome, nausea, or dizziness within the postoperative 48 hours. At 24 hours after surgery, numbness was observed in 1 patient in the I.V. group and 2 patients in the perineural group. Motor block was found in 9 patients in the I.V. group and 11 patients in the perineural group within 24 hours after surgery. Full recovery occurred by the next day in all cases.

**Table 2:**
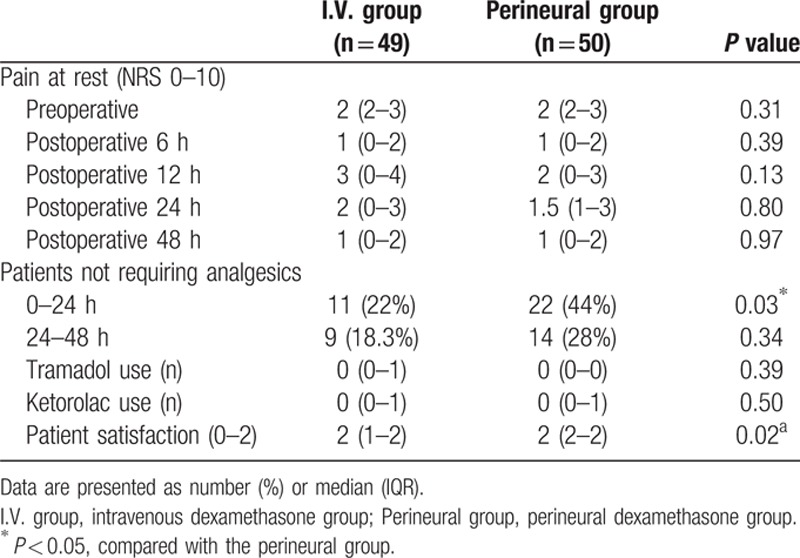
Postoperative outcomes related to pain.

**Table 3:**
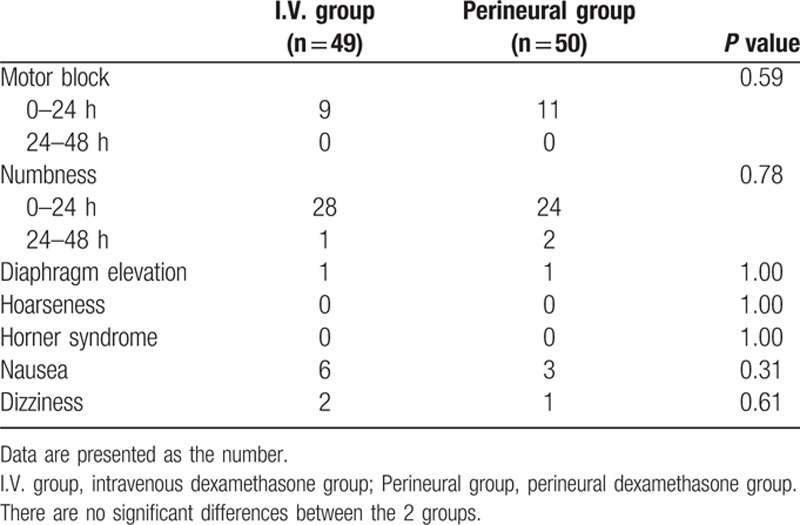
Block characteristics and adverse effects.

Glucose values, presented in mg dL^−1^, are shown in Table 4. There was a significant increase in mean postoperative blood glucose values in both groups compared with baseline values (*P* < 0.001). The glucose value changes (mean ± SD) were 9.6 ± 20.0 in the I.V. group and 7.4 ± 14.1 in the perineural group. There were no significant differences between the 2 groups in baseline (*P* = 0.84) or postoperative blood glucose values (*P* = 0.49).

**Table 4:**
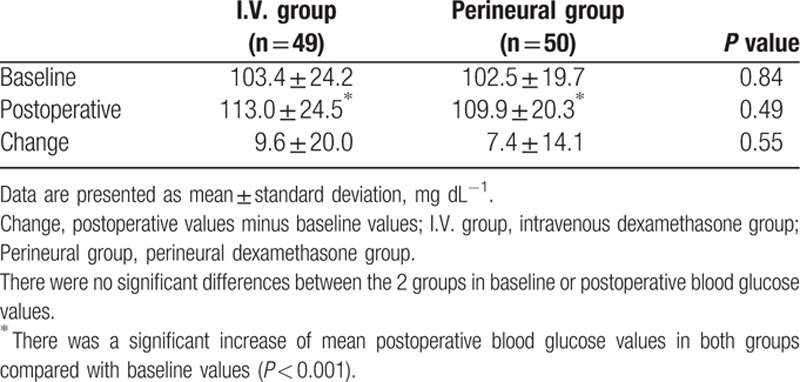
The changes of blood glucose values.

## 4 Discussion

The meaningful finding of the present study was that perineural dexamethasone significantly prolonged the analgesic duration of SISB compared with I.V. dexamethasone. Previous studies have reported that I.V. dexamethasone and perineural dexamethasone similarly prolong the duration of analgesia with SISB.^[5,6]^ However, previous studies do not prove the equivalence of I.V. dexamethasone to perineural dexamethasone; because the previous reports were not noninferiority trials, the sample sizes were too small to find any meaning pertaining to the route of administration of dexamethasone.

Perineural dexamethasone improves postoperative pain outcomes^[9,10]^ and prolongs the effects of BPB^[4]^ when given as an adjunct to local anesthetics. Dexamethasone was found to increase the mean analgesic duration by 233 minutes when injected with short- or medium-term action local anesthetics.^[11]^ The optimal dose of dexamethasone as an adjunct to SISB is debatable. Perineural dexamethasone has been demonstrated to have dose-dependent effects on the duration of SISB analgesia; the inclusion of dexamethasone 2.5, 5.0, and 7.5 mg resulted in significant increases in time to the first analgesic request by factors of 1.6, 2.2, and 1.8, respectively.^[7]^ Meanwhile, meta-regression did not show an interaction between dose of perineural dexamethasone (4–10 mg) and duration of analgesia.^[11]^ The authors reported clinically meaningful prolongation of the analgesic duration with perineural dexamethasone 5 mg in a previous study,^[7]^ so we conducted SISB with 5 mg of perineural dexamethasone.

The mechanism of the effect of dexamethasone as an adjuvant to local anesthetics is unclear. It has been described as a direct effect of glucocorticoid on nerve conduction or a direct blockade of transmission in nociceptive C fibers, along with anti-inflammatory effects.^[12]^ However, dexamethasone did not affect either the A- or the C-wave of compound action potentials. It has been suggested that dexamethasone influences the actions of local anesthetics via indirect mechanisms.^[13]^

Systemic dexamethasone has been used for an effective adjunct in multimodal strategies for preventing postoperative nausea and vomiting, as well as for reducing postoperative pain and opioid consumption.^[2,3,14]^ The present study demonstrated that perineural dexamethasone 5 mg is more effective than I.V. dexamethasone 5 mg with respect to the analgesic duration of SISB, and I.V. dexamethasone 5 mg also prolonged analgesic duration compared with the no adjuvant group of a previous report.^[7]^ However, Kawanishi and co-workers^[15]^ demonstrated that perineural but not I.V. administration of dexamethasone 4 mg significantly prolonged the duration of effective postoperative analgesia resulting from SISB with 20 mL of ropivacaine 0.75%. Several studies have reported conflicting results concerning the route of administration and effects of dexamethasone in nerve block. With respect to BPB, I.V. dexamethasone and perineural dexamethasone were found to similarly prolong analgesic duration.^[5,6,15]^ In contrast, I.V. dexamethasone and perineural dexamethasone had only a minor analgesic enhancing effect in sciatic nerve block.^[16,17]^

There are no data regarding the equivalent dose of I.V. dexamethasone compared to perineural dexamethasone for SISB. A meta-analysis showed that high-dose I.V. dexamethasone (>0.1 mg kg^−1^) is more effective in multimodal strategies for reducing postoperative pain than low-dose I.V. dexamethasone (<0.1 mg kg^−1^).^[2]^ I.V. dexamethasone 10 mg was more potent than I.V. dexamethasone 2.5 mg in the prolongation of analgesic duration. Furthermore, I.V. dexamethasone 1.25 mg also extended analgesic duration.^[18]^ Williams and colleagues^[19]^ have insisted that identical doses of dexamethasone administered intravascularly versus perineurally have inherently different analgesic properties. They have suggested that concurrent administration of dexamethasone via I.V. and perineural routes has great potential for both antiemesis and analgesia.

When prolonging the block, the patient-controlled continuous interscalene brachial plexus block is also effective.^[20–22]^ Despite of decreased the failure rate and the complication,^[23]^ the continuous interscalene brachial plexus block is still underused. Fredrickson and colleagues^[24]^ reported that the complications limited to mild dyspnea, hoarseness, and dysphagia but brachial plexus neuropathy^[25]^ or catastrophic intrathecal malpositioning^[26]^ can occurred. Rebound pain, which means a sudden increase in pain intensity 8 hours after the surgery accompanied by wearoff of the SISB,^[27]^ is shortcomings of SISB. In the present study, NRS at postoperative 6 hours, 12 hours were 1,3 in the I.V. group and 1,2 in the perineural group, respectively. The intensity of rebound pain was inferior to moderate pain in the participants. Moreover, the number of the patients not requiring analgesics was 22% in the I.V. group and 44% in the perineural group. Thus, we expect that the concurrent administration of dexamethasone improves the quality of postoperative analgesia alleviating the rebound pain for arthroscopic shoulder surgery without the possibility of continuous interscalene brachial plexus block-related complication.

Another potential factor influencing analgesic duration is the volume of local anesthetics. Previous researchers have used 30 mL of 0.5% bupivacaine or ropivacaine with dexamethasone 8 mg or 10 mg.^[5,6,8]^ However, in the present study, we performed SISB using 12 mL of 0.5% ropivacaine with dexamethasone 5 mg. The large doses of bupivacaine and the influence of epinephrine may have concealed the true effects of the dexamethasone.^[6]^ As ultrasound guidance has been standardized, a low volume of local anesthetics has been recommended.^[28]^ Thus, we effectively prevent side effects, including diaphragm elevation, hoarseness, and Horner syndrome.

We found a significant increase in mean postoperative blood glucose values in both groups relative to the values prior to dexamethasone administration. In epidural or intra-articular steroid injection using triamcinolone acetate 40 mg, fasting plasma glucose levels were significantly higher 1 day after injection but restored to baseline 7 days after injection. The mean increases in blood glucose levels after epidural injection were 15.6 mg dL^−1^ in non-DM patients and 13.5 mg dL^−1^ in DM patients at 1 day after injection.^[29]^ With SISB, an increase was observed in mean blood glucose concentrations in groups receiving 10 mg of dexamethasone intravenously or via the perineural route.^[5]^ In the present study, there were also no differences in the changes in blood glucose values between the 2 groups. With dexamethasone, the dose and the route of administration may influence the blood glucose level. We suggest that dexamethasone 5 mg can be used without significant differences in blood glucose control whether administered by I.V. or by the perineural route.

The present study had several limitations. First, we did not monitor the duration of motor and sensory block, and we focused on the duration of analgesia only. Second, we did not assess long-term outcomes, including neurological complications. However, any observed neurological symptoms resolved completely by 48 hours after surgery in every patient, and on the day of discharge and on day 14 of follow-up, no patient showed neurological complications. Third, we did not evaluate long-term changes in blood glucose values; we assessed the values only twice, before and after the surgery. Further studies are warranted to determine the most effective guidelines for I.V. or perineural dexamethasone as an effective component of multimodal analgesia with minimal side effects.

In conclusion, we have demonstrated that perineural dexamethasone 5 mg significantly prolonged the duration of analgesia compared with I.V. dexamethasone 5 mg. Dexamethasone via both routes is an effective adjunct for prolonging the duration of analgesia of BPB for arthroscopic surgery. We suggest that 5 mg of perineural dexamethasone is effective in multimodal analgesia for arthroscopic shoulder surgery.
